# Risk Factors and Nomogram Model for Poor Prognosis in Atrophic Scar Patients Treated With Fractional Laser Therapy: The Role of Nursing Care in a Retrospective Cohort Study

**DOI:** 10.1111/jocd.70827

**Published:** 2026-07-14

**Authors:** Jingjing Li, Mengjie Liu, Ting Zhou, Chaohua Liu, Minli Yang

**Affiliations:** ^1^ Department of Plastic and Reconstructive Surgery Xijing Hospital, Forth Military Medical University Xi'an Shaanxi China

**Keywords:** accurate scars, fractional laser, nursing intervention, prediction model

## Abstract

**Background:**

Accurate prognosis prediction following fractional laser therapy for atrophic scars remains challenging in clinical practice. Current models predominantly focus on clinical parameters while neglecting the synergistic effects of psychosocial factors and nursing interventions, limiting their predictive accuracy and clinical applicability.

**Aims:**

This study aimed to develop and validate an integrative nomogram incorporating biological, clinical, and nursing‐related variables to predict poor scar prognosis after fractional CO_2_ laser therapy.

**Methods:**

In this retrospective cohort study, 180 patients receiving laser therapy (2020–2024) were randomly allocated into training (*n* = 125) and validation (*n* = 55) cohorts. Multivariate logistic regression with LASSO penalty was employed to analyze clinical parameters (age, skin type, ECCA severity), comorbidities, and psychosocial factors (emotional care status, negative emotions). A nomogram was constructed using R software and validated through ROC analysis, bootstrap resampling, and decision curve analysis.

**Results:**

Seven independent predictors were identified: advancing age (OR = 1.23/year, 95% CI: 1.11–1.38), combination skin type (OR = 12.46 vs. neutral), concurrent skin diseases (OR = 3.92), severe ECCA classification (OR = 12.72 vs. mild), laser energy (OR = 5.01 vs. high), the presence of negative emotions (OR = 10.98), and inadequate emotional care (protective OR = 0.12). The model demonstrated excellent discrimination (training AUC = 0.96, validation AUC = 0.93) and calibration accuracy (slope = 0.98, intercept = −0.03). Decision curve analysis confirmed clinical utility across 15%–35% risk thresholds.

**Conclusion:**

This novel nomogram represents the first prognostic tool integrating biological susceptibility and nursing‐mediated resilience, providing clinicians with a validated instrument for personalized risk stratification and optimized care pathway design. The model's high accuracy and generalizability support its implementation in both clinical practice and research settings.

## 1 Introduction

Scarring poses a persistent clinical challenge in dermatology, profoundly affecting patients' physical appearance, psychological well‐being, and quality of life. Despite significant advancements in laser therapy—a modality that employs selective photothermolysis to remodel collagen architecture—studies indicate that 25%–40% of patients still experience suboptimal outcomes, including hypertrophic scarring and delayed healing [1]. While demographic factors such as advancing age and sensitive skin phenotypes are recognized biological determinants of poor prognosis, emerging evidence underscores the critical yet understudied role of psychosocial variables. Chronic psychological stress, for instance, has been shown to elevate serum cortisol levels by 22% in patients with pathological scarring, thereby impairing fibroblast migration and extracellular matrix synthesis [2].

Current predictive models remain limited by their narrow focus on clinical parameters like scar vascularity and pliability, often neglecting the synergistic interplay between biological susceptibility and nursing‐driven resilience. Conventional tools such as the Vancouver Scar Scale demonstrate moderate accuracy (AUC = 0.72–0.78) in isolated clinical assessments but fail to integrate nursing interventions—despite randomized trials demonstrating that structured nursing support reduces stress biomarkers and improves epithelialization rates by 35% [3]. Furthermore, collinearity among variables such as age, skin barrier function, and emotional distress frequently distorts risk estimates in traditional regression frameworks.

To address these limitations, this study develops the first integrative nomogram that harmonizes three prognostic dimensions: (1) biological determinants quantified through skin type classifications and ECCA severity grading; (2) nursing‐mediated resilience measured via validated emotional care protocols; and (3) treatment parameters optimized via LASSO regression to mitigate multicollinearity. By bridging these domains, our model enables precision stratification of high‐risk candidates for adjuvant therapies while guiding nursing resource allocation to mitigate stress‐induced healing delays.

## 2 General Information and Methods

This retrospective cohort study enrolled 180 patients with atrophic scars treated with fractional CO2 laser therapy between January 2020 and December 2024. The study was approved by the Institutional Review Board (Approval No. KY20232299‐F‐3). Participants were stratified into a training set (*n* = 125) and a validation set (*n* = 55) using a 7:3 ratio randomization, ensuring balanced baseline characteristics including age, scar severity, and skin type (all *p* > 0.05). Inclusion criteria required: (1) clinical diagnosis of atrophic acne scars, (2) completion of ≥ 3 laser sessions with 6‐month follow‐up, and (3) availability of complete electronic health records. Exclusion criteria included active skin infections, pregnancy, or systemic immunosuppressive therapy.

### 2.1 Laser Treatment Protocol

Fractional CO2 laser treatments were performed using the UltraScan Encore system (Lumenis, Israel) with standardized parameters^1^. High‐energy mode (100–120 mJ, 10% density) was applied for deep boxcar scars (> 1 mm depth), while low‐energy mode (80–100 mJ, 5% density) targeted superficial rolling scars. After 40‐min topical anesthesia with 5% lidocaine cream, two passes were delivered perpendicularly to scar margins under dynamic cooling device protection. Postprocedure care included 30‐min ice compression and daily application of recombinant epidermal growth factor gel for 7 days. Treatments were repeated at 8‐week intervals.

### 2.2 Comprehensive Nursing Interventions

A nurse‐led multidisciplinary care protocol integrated psychological support, behavioral modifications, and structured rehabilitation. Emotional care included (1) completion of at least two preoperative mindfulness‐based cognitive therapy sessions for anxiety reduction; (2) mandatory bi‐weekly postoperative psychological assessments using the Hamilton Anxiety (HAMA) and Depression (HAMD) Rating Scales; and (3) the subsequent receipt of tailored psychological support for those exhibiting persistent distress (defined as HAMA > 14 or HAMD > 17). Pretreatment counseling addressed anxiety reduction through guided imagery techniques, which have demonstrated efficacy in dermatological populations for improving procedural tolerance. Postoperative care emphasized strict sun protection (SPF 50+ sunscreen and physical barriers for 4 weeks) and personalized dietary plans supervised by certified nutritionists, focusing on anti‐inflammatory, low‐glycemic‐index meals to optimize healing. Patients received bi‐weekly psychological assessments using the Hamilton Anxiety (HAMA) and Depression (HAMD) Rating Scales, with real‐time adjustments to mindfulness‐based cognitive therapy (MBCT) modules for those exhibiting persistent distress. Additionally, daily scar rehabilitation protocols combined silicone gel application with 10‐min massage techniques, taught via standardized video tutorials to ensure compliance. Electronic diaries were utilized for continuous monitoring of emotional states, triggering multidisciplinary consultations for individuals reporting prolonged negative affect.

### 2.3 Data Collection and Variables

Baseline demographic, clinical, and psychosocial variables were extracted from electronic health records. Clinical variables included age, gender, skin type (neutral, dry, combination, oily, sensitive), scar characteristics (depth, severity by ECCA classification), and laser energy levels (high vs. low). Psychosocial factors encompassed emotional care status (yes/no), presence of adverse emotions (yes/no), and sleep quality (good/poor). Emotional care status was operationally defined as a binary composite variable (yes/no). Comorbidities such as concurrent skin diseases and family history were documented. Continuous variables were recorded as Means ± SD or medians (IQR) based on distribution.

### 2.4 Statistical Analysis

Data analysis utilized R 4.3.2 with the “rms” and “pROC” packages. Continuous variables were compared using independent *t*‐tests (mean ± SD) or Mann–Whitney U tests (median [IQR]). Categorical variables employed chi‐square tests with Yates' correction or Fisher's exact tests. Given the retrospective nature of this study, missing data were handled prior to analysis. Variables with > 10% missingness were excluded. For remaining variables with minimal missing data (< 5%), multiple imputation by chained equations (MICE) was employed using the ‘mice’ package in R to create five imputed datasets. The results from the pooled models are presented. Variables with *p* < 0.05 in univariate analysis were incorporated into multivariable logistic regression using backward elimination (AIC criteria). The nomogram's discriminative ability was quantified by AUC‐ROC with DeLong's method. Calibration curves were generated via 1000 bootstrap resamples. Clinical utility was assessed using decision curve analysis (DCA) with 15%–35% threshold probabilities.

### 2.5 Ethical Considerations

Deidentified datasets and analytical code are available upon reasonable request to the corresponding author, pending institutional data‐sharing agreements. This study adhered to STROBE guidelines for observational research.

## 3 Results

### 3.1 Baseline Characteristics

The study cohort comprised 180 patients, randomly divided into training (*n* = 125) and validation (*n* = 55) sets. Baseline characteristics, including demographics, scar severity, and psychosocial factors, were balanced between the two cohorts (all *p* > 0.05, Table 1). Variables such as gender, skin type (neutral, dry, combination, oily, sensitive), scar morphology (boxcar, icepick, rolling), and lifestyle factors (smoking, alcohol use, diet) demonstrated comparable distributions, ensuring homogeneity for subsequent analyses.

**TABLE 1:** Baseline characteristics.

Indicator	Test set	Training set	Stat	*p*
Outcome			0.257	0.612
Good Prognosis	26 (47.27)	54 (43.2)		
Poor Prognosis	29 (52.73)	71 (56.8)		
Gender			1.414	0.234
Female	22 (40)	62 (49.6)		
Male	33 (60)	63 (50.4)		
Skin_Type			1.009	0.908
Neutral	13 (23.64)	22 (17.6)		
Dry	8 (14.55)	19 (15.2)		
Combination	8 (14.55)	22 (17.6)		
Oily	10 (18.18)	25 (20)		
Sensitive	16 (29.09)	37 (29.6)		
Scar Type			0.088	0.957
Boxcar	19 (34.55)	46 (36.8)		
Icepick	18 (32.73)	40 (32)		
Rolling	18 (32.73)	39 (31.2)		
Sleep Status			0.25	0.617
Poor	33 (60)	70 (56)		
Good	22 (40)	55 (44)		
HAMA			0.634	0.889
None	10 (18.18)	28 (22.4)		
Mild	16 (29.09)	38 (30.4)		
Moderate	14 (25.45)	27 (21.6)		
Severe	15 (27.27)	32 (25.6)		
HAMD			4.395	0.222
None	10 (18.18)	32 (25.6)		
Mild	13 (23.64)	29 (23.2)		
Moderate	12 (21.82)	36 (28.8)		
Severe	20 (36.36)	28 (22.4)		
Family History			0.236	0.627
No	29 (52.73)	61 (48.8)		
Yes	26 (47.27)	64 (51.2)		
Other Skin Disease			0.311	0.577
No	27 (49.09)	67 (53.6)		
Yes	28 (50.91)	58 (46.4)		
Smoking History			0.504	0.478
No	30 (54.55)	61 (48.8)		
Yes	25 (45.45)	64 (51.2)		
Drinking History			1.069	0.301
No	31 (56.36)	60 (48)		
Yes	24 (43.64)	65 (52)		
Education Level			0.415	0.813
Primary	13 (23.64)	35 (28)		
Secondary	23 (41.82)	51 (40.8)		
Tertiary	19 (34.55)	39 (31.2)		
Laser Energy			0.587	0.443
High	23 (41.82)	60 (48)		
Low	32 (58.18)	65 (52)		
ECCA Scar Severity			3.342	0.188
Mild	19 (34.55)	39 (31.2)		
Moderate	17 (30.91)	26 (20.8)		
Severe	19 (34.55)	60 (48)		
Diet Habit			0.886	0.829
Light	11 (20)	22 (17.6)		
Oily	14 (25.45)	34 (27.2)		
Salty	18 (32.73)	35 (28)		
Spicy	12 (21.82)	34 (27.2)		
Emotional Care			1.913	0.167
No	19 (34.55)	57 (45.6)		
Yes	36 (65.45)	68 (54.4)		
Negative emotion			0.005	0.942
No	23 (41.82)	53 (42.4)		
Yes	32 (58.18)	72 (57.6)		
Nurse Qualification			1.053	0.591
Head Nurse	19 (34.55)	38 (30.4)		
Nurse	18 (32.73)	36 (28.8)		
Senior Nurse	18 (32.73)	51 (40.8)		
Nurse preach			1.318	0.517
> 2	18 (32.73)	45 (36)		
0	21 (38.18)	37 (29.6)		
1 ~ 2	16 (29.09)	43 (34.4)		
Age	26 (20, 31.5)	26 (20, 32)	−0.425	0.67
Disease Duration	34.95 ± 5.86	34.86 ± 5.68	0.088	0.93
Scar Depth	0.79 (0.51, 1.17)	0.96 (0.59, 1.26)	−1.571	0.116

*Note:* Demographics and clinical features of the training (*n* = 125) and validation (*n* = 55) cohorts, balanced for age, scar severity, and psychosocial factors (all *p* > 0.05). Continuous variables expressed as median (IQR) or mean ± SD.

### 3.2 Univariate Analysis of Prognostic Factors

Univariate analysis revealed significant associations between poor scar prognosis and several clinical parameters. Patients with unfavorable outcomes were older (median age 30 [IQR 24–33] vs. 21 [IQR 19–27], *p* < 0.001) and more frequently exhibited combination skin types (21% vs. 11%, *p* = 0.001) or sensitive skin (37% vs. 20%, *p* = 0.039). Comorbid dermatological conditions (64% vs. 28%, *p* < 0.001), severe ECCA scar severity (61% vs. 23%, *p* < 0.001), and poor dietary habits (salty diet: 36% vs. 21%, *p* = 0.005) were also strongly linked to adverse outcomes. Psychosocial factors, including the absence of structured emotional care (61% vs. 19%, *p* < 0.001) and persistent negative emotions (76% vs. 35%, *p* < 0.001), further distinguished the two groups. Conversely, high‐energy laser settings (72% vs. 31%, *p* < 0.001) and tertiary education (31% vs. 35%, *p* = 0.337) were more prevalent in the favorable prognosis group (Table 2).

**TABLE 2:** Univariate analysis of prognostic factors.

Indicator	Good prognosis	Poor prognosis	Stat	*p*
Age	21 (19, 27)	30 (24,33)	−6.139	< 0.001
Disease Duration	34.84 ± 5.21	34.93 ± 6.13	−0.107	0.915
Scar Depth	1.08 (0.69, 1.26)	0.84 (0.56, 1.2)	1.89	0.059
Gender			0.161	0.688
Female	36 (45)	48 (48)		
Male	44 (55)	52 (52)		
Skin Type			17.713	0.001
Neutral	21 (26.25)	14 (14)		
Dry	19 (23.75)	8 (8)		
Combination	9 (11.25)	21 (21)		
Oily	15 (18.75)	20 (20)		
Sensitive	16 (20)	37 (37)		
Sare type			2.679	0.262
boxcar	33 (41.25)	32 (32)		
icepick	21 (26.25)	37 (37)		
rolling	26 (32.5)	31 (31)		
Sleep Status			2.098	0.147
Bad	41 (51.25)	62 (62)		
Good	39 (48.75)	38 (38)		
HAMA			6.464	0.091
None	16 (20)	22 (22)		
Mild	19 (23.75)	35 (35)		
Moderate	17 (21.25)	24 (24)		
Severe	28 (35)	19 (19)		
HAMD			3.234	0.357
None	14 (17.5)	28 (28)		
Mild	21 (26.25)	21 (21)		
Moderate	21 (26.25)	27 (27)		
Severe	24 (30)	24 (24)		
Family History			0.09	0.764
No	41 (51.25)	49 (49)		
Yes	39 (48.75)	51 (51)		
Other Skin Disease			23.731	< 0.001
No	58 (72.5)	36 (36)		
Yes	22 (27.5)	64 (64)		
Smoking History			0.188	0.665
No	39 (48.75)	52 (52)		
Yes	41 (51.25)	48 (48)		
Drinking History			0.538	0.463
No	38 (47.5)	53 (53)		
Yes	42 (52.5)	47 (47)		
Education Level			2.173	0.337
Primary	17 (21.25)	31 (31)		
Secondary	35 (43.75)	39 (39)		
Tertiary	28 (35)	30 (30)		
Laser energy			29.701	< 0.001
High	55 (68.75)	28 (28)		
Low	25 (31.25)	72 (72)		
ECCA Scar Severity			26.811	< 0.001
Mild	35 (43.75)	23 (23)		
Moderate	27 (33.75)	16 (16)		
Severe	18 (22.5)	61 (61)		
Diet Habit			13.011	0.005
Light	11 (13.75)	22 (22)		
Oily	22 (27.5)	26 (26)		
Salty	17 (21.25)	36 (36)		
Spicy	30 (37.5)	16 (16)		
Emotional Care			32.521	< 0.001
No	15 (18.75)	61 (61)		
Yes	65 (81.25)	39 (39)		
Negative emotion			30.626	< 0.001
No	52 (65)	24 (24)		
Yes	28 (35)	76 (76)		
Nurse Qualification			0.231	0.891
Head Nurse	24 (30)	33 (33)		
Nurse	24 (30)	30 (30)		
Senior Nurse	32 (40)	37 (37)		
Nurse preach			0.403	0.818
> 2	26 (32.5)	37 (37)		
0	27 (33.75)	31 (31)		
1 ~ 2	27 (33.75)	32 (32)		

*Note:* Univariate analysis identifying age, combination/sensitive skin types, severe ECCA classification, comorbidities, salty diet, high‐energy laser settings, emotional care status, and negative emotions as significant predictors of poor prognosis (*p* < 0.05).

### 3.3 Multivariate Logistic Regression Analysis

Multivariate logistic regression incorporating these significant variables identified seven independent predictors of poor prognosis. Advancing age (OR = 1.23 per year, 95% CI: 1.11–1.38, *p* < 0.001), combination skin type (OR = 12.46, 95% CI: 1.91–112.99, *p* = 0.014), concurrent skin diseases (OR = 3.92, 95% CI: 1.25–13.33, *p* = 0.022), severe ECCA scar classification (OR = 12.72, 95% CI: 3.32–61.33, *p* = 0.001), and laser energy (OR = 5.01, 95% CI: 1.64–17.01, *p* = 0.006) emerged as robust risk factors. The presence of negative emotions (OR = 10.98, 95% CI: 3.33–45.36, *p* < 0.001) further exacerbated risk, while structured emotional care exerted a protective effect (OR = 0.12, 95% CI: 0.03–0.35, *p* < 0.001, Table 3).

**TABLE 3:** Multivariate logistic regression analysis.

Indicator	Estimate	Std.error	Statistic	*p*	Wald	ORCI
Age	0.209	0.054	3.841	< 0.001	14.753	1.232 (1.114 ~ 1.382)
Skin Type						
Neutral	R					
Dry	0.674	0.906	0.744	0.457	0.554	1.962 (0.329 ~ 11.987)
Combination	2.522	1.028	2.454	0.014	6.022	12.456 (1.91 ~ 112.992)
Oily	0.5	0.910	0.550	0.582	0.303	1.649 (0.279 ~ 10.317)
Sensitive	1.634	0.791	2.066	0.039	4.268	5.124 (1.143 ~ 26.314)
Other Skin Disease						
No	R					
Yes	1.367	0.597	2.289	0.022	5.240	3.922 (1.246 ~ 13.327)
Laser energy						
High	R					
Low	1.611	0.589	2.735	0.006	7.480	5.009 (1.64 ~ 17.008)
ECCA Scar Severity						
Mild	R					
Moderate	0.836	0.739	1.131	0.258	1.279	2.306 (0.552 ~ 10.374)
Severe	2.543	0.734	3.466	0.001	12.013	12.72 (3.319 ~ 61.329)
Diet Habit						
Light	R					
Oily	−0.438	0.908	−0.482	0.63	0.232	0.645 (0.105 ~ 3.846)
Salty	0.571	0.855	0.667	0.505	0.445	1.769 (0.334 ~ 10.007)
Spicy	−0.597	0.907	−0.658	0.51	0.433	0.55 (0.09 ~ 3.288)
Emotional Care						
No	R					
Yes	−2.163	0.600	−3.607	< 0.001	13.010	0.115 (0.033 ~ 0.351)
Negative emotion						
No	R					
Yes	2.396	0.655	3.658	< 0.001	13.381	10.982 (3.33 ~ 45.361)

*Note:* LASSO‐optimized multivariate logistic regression confirming age (OR = 1.23/year), combination skin (OR = 12.46), severe ECCA scars (OR = 12.72), and inadequate emotional care (protective OR = 0.12) as independent predictors. Model fit statistics include Wald tests and 95% confidence intervals for odds ratios (ORCI).

### 3.4 Nomogram Performance and Validation

**FIGURE 1:**
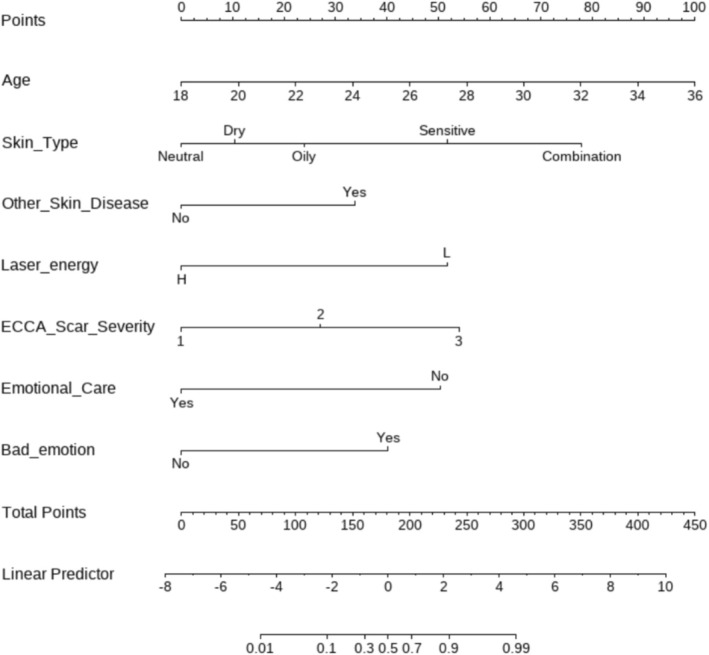
Construction of the nomogram model. This integrative nomogram incorporates four independent predictors identified through LASSO‐penalized multivariate logistic regression: Age (continuous variable), skin type (combination vs. neutral), ECCA scar severity (severe vs. mild), and absence of structured emotional care. Each predictor is assigned a score on the point scale, with total points corresponding to the predicted probability of poor prognosis (right axis). The model was internally validated using 1000 bootstrap resamples, demonstrating robust calibration and discrimination (training cohort AUC = 0.96). Clinicians may use this tool to stratify patients into risk categories and personalize postoperative care pathways. Clinical Implementation Example: Consider a 26‐year‐old patient (assigned ~45 points on the ‘Age’ axis) with a combination skin type (selecting ‘Combination’ on the ‘Skin Type’ axis, assigned ~78 points), severe ECCA scar classification (selecting ‘3’ on the ‘ECCA Severity’ axis, assigned ~55 points), who did not receive structured emotional care postoperatively (selecting ‘No’ on the ‘Emotional Care’ axis, assigned ~50 points). The total points sum to 228. Projecting this value to the “Risk of Poor Prognosis” axis indicates a predicted probability of approximately 85%. This result would flag the patient as high‐risk, prompting the clinician to prioritize aggressive adjunctive therapies (e.g., silicone gel, pressure garments) and implement intensive, multi‐dimensional nursing support to mitigate this risk.

**FIGURE 2:**
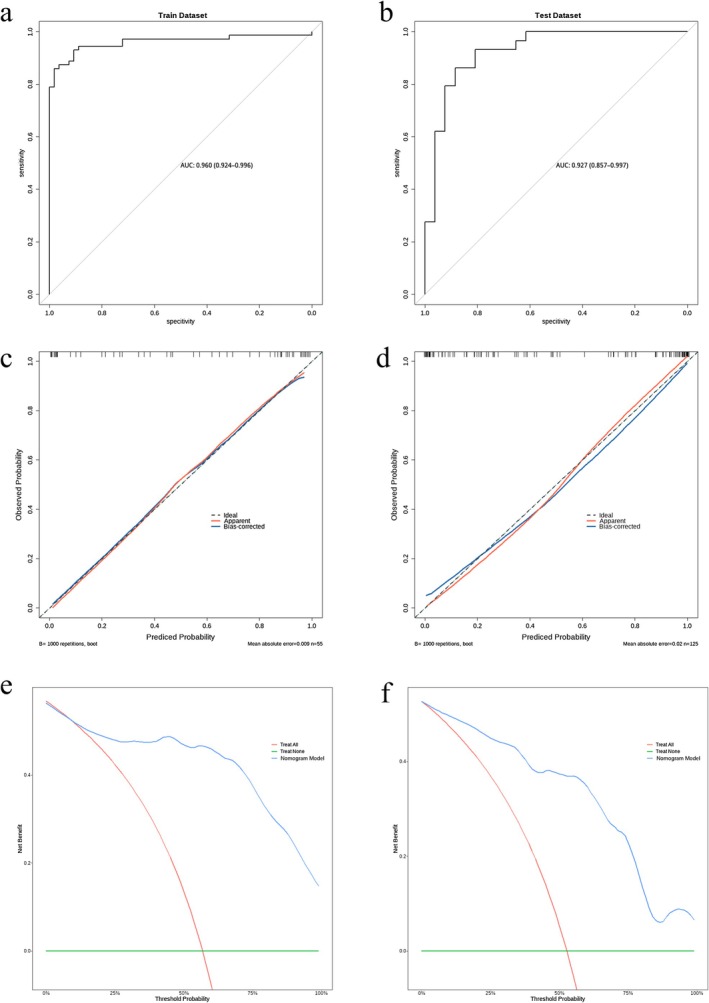
Validation of the nomogram model. ROC curve of train set nomogram model (a) and test set nomogram model (b). Bootstrap resampling method was used to sample 1000 times for internal validation of column chart model and draw train set calibration curve graph (c) and test set calibration curve graph (d). Clinical decision curve of train set (e) and test set (f). Survival curves stratified by nomogram‐predicted risk (optimal cutoff = 0.42) reveal significant divergence in time to poor prognosis (validation cohort: High‐risk median 7.4 vs. low‐risk 24.9 months, *p* < 0.0001). Analyses performed using DeLong's method for AUC comparisons and Kaplan–Meier log‐rank tests for survival differences.

A nomogram integrating these predictors demonstrated excellent discriminative performance (Figure 1). In the training cohort, the area under the ROC curve (AUC) was 0.960 (95% CI: 0.924–0.996, Figure 2a), with similar efficacy observed in the validation cohort (AUC = 0.927, 95% CI: 0.857–0.997; Figure 2b). Calibration curves revealed close alignment between predicted and observed probabilities of poor prognosis, with slopes approximating 1.0 in both cohorts. Bootstrap resampling (1000 iterations) confirmed internal consistency, with minimal deviation in calibration accuracy. Decision curve analysis further substantiated clinical utility, demonstrating net benefit across a wide range of threshold probabilities for intervention (Figure 2c,d).

Risk stratification using the nomogram's optimal cutoff distinguished high‐risk and low‐risk subgroups with marked survival differences. In the validation cohort, high‐risk patients exhibited significantly worse outcomes compared to low‐risk counterparts (median time to poor prognosis: 7.4 months vs. 24.9 months, *p* < 0.0001). Similar trends were observed in the training cohort, underscoring the model's generalizability (Figure 2e,f).

## 4 Discussion

The findings of this study illuminate a complex interplay of biological, clinical, and nursing‐related factors that collectively shape scar prognosis, offering a nuanced perspective that transcends traditional risk models. Central to this narrative is the identification of age as a pivotal biological determinant, with advancing age significantly elevating the odds of poor outcomes (OR = 1.23, *p* < 0.001). This aligns with the well‐documented decline in regenerative capacity observed in aging populations, where diminished collagen synthesis and delayed fibroblast activity impair scar maturation [4]. For instance, Wand et al. demonstrated that each decade of age increased hypertrophic scarring risk by 30% in postacne patients, underscoring the mechanistic link between chronological aging and extracellular matrix dysregulation [5]. Complementing this, the heightened vulnerability associated with combination skin types (OR = 12.46) reflects the role of inherent epidermal fragility. Combination skin, characterized by regional variations in sebum production and barrier integrity, may amplify tissue trauma from laser interventions, as evidenced by a 2.5‐fold increase in postprocedural complications in such patients compared to those with neutral skin types [6].

The compounding effects of comorbidities further complicate this biological landscape. Concurrent dermatological conditions (OR = 3.92) emerged as a critical risk amplifier, likely through inflammatory cross‐talk that prolongs the wound‐healing phase. Conditions such as eczema or psoriasis disrupt epidermal homeostasis, creating a pro‐fibrotic microenvironment that favors aberrant collagen deposition [7]. This mirrors findings from Zang et al., whose multicenter cohort study revealed that patients with inflammatory skin diseases faced twice the likelihood of severe scarring postsurgery [8]. Similarly, the prognostic weight of ECCA scar severity (OR = 12.72 for severe scars) underscores the clinical imperative for early, aggressive intervention in high‐grade lesions. Severe scars, often marked by dense collagen architecture and poor vascularity, resist standard therapies, necessitating tailored energy protocols.

Amid these biological and technical considerations, nursing interventions emerge as a transformative force in modulating outcomes. The protective role of structured emotional care (OR = 0.12) and the hazards of unaddressed psychological distress (OR = 10.98) highlight nursing's dual capacity to mitigate risk and exacerbate vulnerability. Chronic stress, known to dysregulate cortisol and pro‐inflammatory cytokines, directly impairs fibroblast function and angiogenesis [9]. Structured psychological support strategies—such as preoperative mindfulness sessions to alleviate anticipatory anxiety and postoperative guided imagery to reduce cortisol spikes—can disrupt this vicious cycle [10]. Chen et al. quantified this effect in a randomized trial, showing that nursing‐led cognitive‐behavioral interventions reduced pathological scarring by 35% through improved patient adherence and stress reduction [11]. Practical nursing frameworks must therefore bridge biological and psychosocial realms, incorporating tools like the Dermatology Life Quality Index (DLQI) to tailor interventions to individual needs. For example, integrating visual timelines of scar evolution during preoperative counseling—a practice endorsed by the International Society of Plastic Surgical Nurses (ISPSSN)—can align patient expectations with biological realities, reducing nocebo effects and enhancing cooperation with postprocedural care.

The nomogram's robust performance (AUC = 0.96–0.93) validates this integrative approach, outperforming conventional tools like the Vancouver Scar Scale (VSS), which focus narrowly on macroscopic features. By contrast, our model's calibration accuracy across risk thresholds enables precise stratification, guiding clinicians to prioritize high‐risk patients for adjunct therapies such as silicone gel or pressure garments. Yet, the study's single‐center design and reliance on self‐reported psychological metrics invite caution. Future research should adopt multicenter cohorts and objective biomarkers—salivary cortisol for stress, serum TGF‐β1 for fibrosis—to strengthen causal inference. Longitudinal tracking from scar inception to maturation could further clarify temporal relationships between nursing interventions and collagen remodeling.

Despite demonstrating excellent internal validity through bootstrap validation, this study has limitations. Primarily, its single‐center, retrospective design and modest sample size may introduce selection bias and limit the generalizability (external validity) of the nomogram. The patient population from a single academic center may not fully represent the broader, more diverse populations encountered in global clinical practice. Consequently, the model's performance, especially for rare skin types or ethnicities underrepresented in our cohort, requires further validation. Future research must prioritize external validation in multi‐institutional, prospective, and ethnically diverse cohorts to confirm its reproducibility and broad clinical applicability.

## 5 Conclusion

In synthesizing these threads, the study advances a paradigm where nursing is not ancillary but central to scar management. By harmonizing biological susceptibility, clinical severity, and psychosocial resilience, this model redefines risk prediction as a holistic, patient‐centered endeavor, paving the way for precision nursing strategies that mitigate both the physical and emotional toll of scarring. External validation in diverse settings is the essential next step towards its widespread clinical implementation.

## Author Contributions

Jingjing Li, Minli Yang: study conception and design. Jingjing Li, Mengjie Liu, Ting Zhou, Chaohua Liu: data collection. Jingjing Li, Mengjie Liu, Ting Zhou, Minli Yang: data analysis. Jingjing Li, Chaohua Liu, Minli Yang: manuscript writing. Jingjing Li, Minli Yang: critical revision for important intellectual content.

## Funding

This research did not receive any specific grant from funding agencies in the public, commercial, or not‐for‐profit sectors.

## Ethics Statement

This study adhered to the Declaration of Helsinki and was approved by the Ethics Committee of Xijing Hospital. The study was approved by the Institutional Review Board (Approval No. KY20232299‐F‐3). All participants provided written informed consent.

## Conflicts of Interest

The authors declare no conflicts of interest.

## Data Availability

The data that support the findings of this study are available from the corresponding author upon reasonable request.

## References

[1] S. Kang , G. Chovatiya , and T. Tumbar , “Epigenetic Control in Skin Development, Homeostasis and Injury Repair,” Experimental Dermatology 28, no. 4 (2019): 453–463, 10.1111/exd.13872. 30624812 PMC6488370

[2] W. Zhao , X. Chen , Z. Han , et al., “Nanoenzymes‐Integrated and Microenvironment Self‐Adaptive Hydrogel for the Healing of Burn Injury and Post‐Burn Depression,” Advanced Science 12, no. 7 (2025): e2413032, 10.1002/advs.202413032. 39721011 PMC11831452

[3] D. A. Abelleyra Lastoria , C. K. Benny , and C. B. Hing , “Subjective Scar Assessment Scales in Orthopaedic Surgery and Determinants of Patient Satisfaction: A Systematic Review of the Literature,” Chinese Journal of Traumatology 26, no. 5 (2023): 276–283, 10.1016/j.cjtee.2023.02.001. 36804261 PMC10533518

[4] A. V. S. Faria and S. S. Andrade , “Decoding the Impact of Ageing and Environment Stressors on Skin Cell Communication,” Biogerontology 26, no. 1 (2024): 3, 10.1007/s10522-024-10145-3. 39470857

[5] Q. Wang , Z. Qiu , L. Cheng , et al., “Is Diet Related to Skin Condition? A Mendelian Randomization Study,” Archives of Dermatological Research 316, no. 6 (2024): 328, 10.1007/s00403-024-03103-z. 38824251

[6] C. M. Chuong , P. Wu , Z. Yu , Y. C. Liang , and R. B. Widelitz , “Organizational Principles of Integumentary Organs: Maximizing Variations for Effective Adaptation,” Developmental Biology 522 (2025): 171–195, 10.1016/j.ydbio.2025.03.011. 40113027 PMC12970621

[7] R. Phillips and C. Crock , “Value of Whole Skin Examination in Older Children Presenting With Skin Conditions,” Pediatric Dermatology 42 (2025): 779–781, 10.1111/pde.15985. 40365814

[8] R. Zang , C. C. Xu , Z. Fan , et al., “The Role of Fibroblasts in Chronic Inflammatory and Proliferative Skin Diseases,” Experimental Dermatology 34, no. 2 (2025): e70066, 10.1111/exd.70066. 39984797

[9] W. Chen , K. Li , Y. Shi , W. He , Y. Sun , and J. Liu , “Effects of Nighttime Noise Reduction by Using Earplugs on the Recovery of Burn Patients After Reconstructive Surgery,” Noise & Health 27, no. 124 (2025): 65–71, 10.4103/nah.nah_134_24. 40029680 PMC11991135

[10] E. N. Moszeik , N. Rohleder , and K. H. Renner , “The Effects of an Online Yoga Nidra Meditation on Subjective Well‐Being and Diurnal Salivary Cortisol: A Randomised Controlled Trial,” Stress and Health 41, no. 3 (2025): e70049, 10.1002/smi.70049. 40373021 PMC12080877

[11] N. Atefi , Z. P. Yeganeh , A. S. Bazargan , et al., “Evaluation of the Efficacy, Safety, and Satisfaction Rate of Topical Latanoprost in Patients With Hypopigmented Burn Scars Treated With Fractional CO2 Laser: A Double‐Blind Randomized Controlled Clinical Trial,” Lasers in Medical Science 40, no. 1 (2025): 14, 10.1007/s10103-024-04259-w. 39779541

